# Close of the Volume

**Published:** 1876-12

**Authors:** 


### Editorial and Miscellaneous.

*Close of the Volume.*—The present number closes the sixth volume of THE SOUTHERN MEDICAL RECORD. We suggest to our readers the propriety of having the volumes bound, as they will find THE RECORD invaluable as a book of reference. By reference to the index of the present volume, and those which have preceded it, it will be seen that the number of items published surpasses that of any journal ever published in the United States, if not in the whole world. Stand by us in the future, friends, and we will promise that our next volume, though smaller in size, will not fall short of an equal number of important and practical items.

Send up your renewals, and procure us one or more new subscribers.

☞ *Clubs.*—Those who will renew their subscriptions and send us four new subscribers on our new basis of $2 00 per annum, will be entitled to his number free of charge, and will be enrolled on our list of *co-laborers.* Our reduced terms ought to procure us a large addition to our list of subscribers.

☞ Parties who wish to renew for 1877 we hope will notify us at once. The subscription price, now reduced to $2 00, need not be forwarded until the reception of the January number, unless preferred; but the notice of renewal is desired in advance, to enable us to know the size of the edition to be issued, and to make the most advantageous arrangements with the publishers.

Address DR. R. C. WORD,
Business Manager.

